# Cervical Cancer Screening Uptake and Predictors Among Women in Jeddah, Saudi Arabia

**DOI:** 10.7759/cureus.24065

**Published:** 2022-04-12

**Authors:** Sultanah F Alsalmi, Sahar S Othman

**Affiliations:** 1 Department of Family Medicine, Faculty of Medicine, King Abdulaziz University, Jeddah, SAU

**Keywords:** family physician, conventional papanicolaou (pap) smear, pap test, pap smear, jeddah, women, predictors, screening, cancer, cervical

## Abstract

Introduction: Cervical cancer is one of the most common cancers among females, contributing to significant mortality and morbidity worldwide. These numbers have significantly decreased since the implementation of cervical cancer screening. Despite that, screening in many countries, including Saudi Arabia, remains suboptimal.

Methods: A cross-sectional study was conducted between May to November 2021 among 385 women aged 21-65 years who live in Jeddah, Saudi Arabia. The data were collected using a four-part online survey: demographic characteristics, cervical cancer screening status, predictors of undergoing cervical cancer screening, and barriers to screening.

Results: Among the 385 women who completed the survey, only around one-third (33.4%) had a Pap smear at some point in their lives. The factors that were found to be significantly associated with the screening status (having a Pap test) in the univariate analysis are increasing age, education level, monthly income, perceived risk of getting cervical cancer, source of information about Pap test, having a family doctor, recommendation by the family doctor to have a Pap test, undergoing a gynecological examination, visiting a gynecologist in the past, history of previous gynecological complaint, and history of abortion. In the multivariable analysis, only four factors were found to be significantly associated with the screening status: age, monthly income, undergoing a gynecological examination in the past, and the recommendation by the family doctor, which by far had the largest effect.

Conclusion: Cervical cancer screening rate is relatively low in the city of Jeddah. The recommendation of a Pap test by the family doctor had the largest impact on screening status. These results support the important role of family physicians in promoting screening tests for preventive healthcare. The results also suggest the need for education programs to promote cervical cancer screening among women in Saudi Arabia.

## Introduction

Cervical cancer is the fourth most common cancer in females [1]. In 2018, approximately 570,000 women were diagnosed with cervical cancer, and approximately 311,000 women died worldwide [1]. The majority of cases occur in women who have not been screened [2]. In 2020, the crude cervical cancer incidence per 100,000 women in Saudi Arabia was 2.4 and the cervical cancer mortality to incidence ratio was 0.5 [1].

The United States (US) Preventive Services Task Force recommends screening for women aged 21-65 years every three years to reduce cervical cancer incidence and mortality [2]. Screening rates in many countries, including Saudi Arabia, remain suboptimal [3-4]. A study in Jeddah in 2009 found that only 16.8% of the women in Jeddah underwent Pap screening [3]. In 2019, a study involving residents of four Gulf countries reported cervical cancer screening rates of 7.6% in Saudi Arabia, 10.6% in Oman, 17.7% in Kuwait, and 28.0% in the United Arab Emirates [4].

Many international studies have investigated the predictors and barriers to cervical cancer screening uptake in various countries. A study in Tehran in 2017 investigating the factors preventing women from a second Pap smear test included negative experiences with the first Pap test; personal barriers, such as inattention to time and inhibiting beliefs; and social barriers, such as a lack of social support [5]. In 2018, a study in Addis Ababa among HIV-positive women found that in addition to lack of knowledge and awareness, the lack of guidelines by the provider was a significant barrier to Pap tests [6]. A study in Peru in 2019 revealed that the barriers to accepting the Pap test were low socioeconomic status, low income, lack of appropriate counseling, and fear or embarrassment during the screening procedure [7]. In 2019, a study in the US concluded that White women and those with a high school education had longer intervals between Pap tests [8]. Furthermore, women who tested positive for human papilloma virus (HPV) were five times more likely to complete a follow-up Pap test compared with those who tested negative [8].

A study in India in 2019 found the following categories of women with poor cervical cancer screening rates: women with a low wealth index, women living in cities, those who had not heard of sexually transmitted infections, and women who do not use a modern method of contraception [9]. In 2020, a study in Uganda reported that screening rates were higher among women who were professional, wealthy, had a high level of knowledge about cervical cancer and screening, had four or more children, and whose main source of information about screening was healthcare providers [10]. Another study in Ethiopia in 2020 found higher educational levels, a history of HIV testing, and high perceived self-efficacy were significant predictors for cervical cancer screening service uptake [11].

The predictors of uptake and barriers to complying with cervical cancer screening recommendations have not been thoroughly investigated on a national scale [12]. According to one study conducted in 2017 in the Al-Hassa region (Saudi Arabia) among secondary school teachers, only 17.2% had been examined for cervical cancer previously [12]. Personal fears were the most significant impediment to screening, followed by healthcare-related factors [12]. Few other national studies have investigated Saudi women's knowledge, attitudes, and practices regarding cervical cancer screening [3-4]. All studies concluded that a lack of awareness was a significant factor in the failure of undergoing a Pap test [3-4].

National studies in the Kingdom of Saudi Arabia show low screening rates [13]. However, predictors of cervical cancer screening uptake among Saudi women have not been fully explored. The purpose of this study was to estimate the prevalence of cervical cancer screening among women in Jeddah, Saudi Arabia, and identify predictors of screening uptake and barriers to adherence to cervical cancer screening recommendations.

## Materials and methods

The study was conducted by researchers from King Abdelaziz University Hospital, Jeddah, Saudi Arabia, who collected the data from the targeted population (women in Jeddah) through an online survey.

Study design and settings

This is a cross-sectional study conducted between May and November 2021 in Jeddah, Saudi Arabia.

Participants

Inclusion criteria were females who reside in Jeddah, aged 21-65 years, who were sexually active at some point in their lives (married, divorced, or widowed), and who volunteered to participate in the study. Anyone who did not meet the inclusion criteria (females <21 years or >65 years, or those who have never been sexually active) were excluded.

Data collection

Participants completed a self-administered survey on a specifically designated platform (Google Forms, Google LLC, Mountain View, California, United States). The survey was constructed after reviewing previously published relevant studies [8-11].and was distributed through different social media platforms targeting the study population. The survey was reviewed independently by two consultants with expertise in the field. It was initially constructed in English, but the authors (who are both bilingual) completed the bidirectional translation, and the survey was available in Arabic and English. A pilot study was conducted among 37 participants (10% of the sample size ) to ensure the validity and consistency of the questionnaire and no changes were made after it.

The first page of the survey included consent to participate in the study. The study aim and target population (eligibility to participate) was stated. Participants were made aware that all answers would be anonymous and confidential. The informed consent provided two options: “yes” for those who agreed to volunteer and fill out the survey, and “no” for those who did not wish to participate. Only those who consented and selected “yes” proceeded to the next page to complete the survey. The survey comprised four sections: demographic characteristics, cervical cancer screening status, predictors of undergoing cervical cancer screening (including knowledge about cervical cancer and Pap smear and gynecological and obstetrical history), and barriers to screening. Demographic data included age, marital status, nationality, area of residence in Saudi Arabia, education, occupation, and monthly income.

Sample size

The sample size was calculated by using the single proportion equation in the Raosoft Sample Size Calculator (Raosoft, Inc., Seattle, Washington, US). Based on the assumption that the rate of cervical cancer screening is 50%, and a margin of error of 5% at the 95% confidence level, the required sample size was 385. The snowball sampling technique was employed, and the survey was distributed online to avoid physical contact during the coronavirus pandemic.

Measures

The primary outcome variable for this study was participating in screening for cervical cancer, defined by answering “yes” to the question: “Have you ever had a Pap test in the past?”. The first questions in the predictors section enquired about knowledge and perceived risk of developing cervical cancer using the following questions:

1. “Have you heard about a disease called cervical cancer?” 

2. “Compared with other women of your age, what do you think your chances of getting cervical cancer are?”

Other questions in this section inquired about knowledge of Pap tests, the source of this information, whether the participant had a family physician, the gender of the family physician, and if the family physician had recommended a Pap test or not. In addition, participants were asked about previous gynecological problems, visits to a gynecologist, undergoing a gynecological examination, the number of children, and history of abortion. Finally, participants who stated that they had not undergone a Pap test were asked to indicate the reason(s).

Data analysis

Statistical analysis was conducted using IBM SPSS Statistics for Windows, Version 20.0 (Released 2011; IBM Corp., Armonk, New York, US). First, descriptive statistics were presented for the whole sample. Qualitative data were presented as frequencies and percentages, while quantitative data were presented as medians and inter-quartile range. Further descriptive statistics were recorded for each of the two groups of the study’s primary outcome: those who had had a Pap test and those who had not. Comparison of each variable between groups was analyzed using a Chi-square test for qualitative variables, and a non-parametric Mann-Whitney U test for quantitative variables (as our data was found to be non-normally distributed).

The association of each predictor with the outcome (having undergone cervical cancer screening) was further tested by conducting univariate binomial regression for each variable. The variables that showed a significant association (p < 0.05) with the outcome (screening status) in the univariate analyses, as well as those with a near significant association (p < 0.1) were entered into a multivariate binary logistic regression model. Bivariate Pearson correlation was used to test the assumption of the multivariate binary regression model. Statistical significance was established at p < 0.05.

Ethical considerations

This study was designed and conducted in accordance with the ethical principles established by the National Committee of Bio and Medical Ethics at King Abdulaziz City for Science and Technology, Saudi Arabia. Ethical approval was obtained on May 6, 2021, from the Biomedical Research Ethics Committee, Faculty of Medicine, King Abdulaziz University, Jeddah, Saudi Arabia (Reference number 295-21).

## Results

As shown in Table 1, 28.8% of the participants were in the age group of 45-54 years, 89.6% were married, 92.4% were Saudis, and 69.1% were residents of the northern region of Jeddah city. Regarding education level, 45.9% were educated to high school or lower. A total of 45.6% of the participants were housewives, and 36.6% had a monthly income of 4000-10000 SR (Saudi Riyal). In terms of knowledge and perceived risk, most of the participants (79.7%) had heard of cervical cancer, and 46.1% reported that their chance of getting cervical cancer was much below average. The rest of the demographic and participants’ characteristics, as well as the other predictors, are described in Table 1.

**Table 1:** Frequency distribution of participants’ characteristics according to their status of cervical cancer screening (had Pap test or not) SR: Saudi Riyal

Variable	Status of cervical cancer screening (Pap test)		Total N = 434 (100%)
	Yes 145 (33.4%)	No 289 (66.6%)	
Age			
21–24 years	4 (0.9%)	34 (7.8%)	38 (8.7%)
25–34 years	29 (6.6%)	85 (19.5%)	114 (26.3%)
35–44 years	41 (9.4%)	83 (19.1%)	124 (28.6%)
45–54 years	55 (12.6%)	70 (16.1%)	125 (28.8%)
55–65 years	16 (3.6%)	17 (3.9%)	33 (7.6%)
Marital status			
Married	129 (29.7%)	260 (59.9%)	389 (89.6%)
Divorced	11 (2.5%)	23 (5.2%)	34 (7.8%)
Widowed	5 (1.1%)	6 (1.3%)	11 (2.5%)
Nationality			
Saudi	137 (31.5%)	264 (60.8%)	401 (92.4%)
Non-Saudi	8 (1.8%)	25 (5.7%)	33 (7.6%)
Area of residence			
South of Jeddah	27 (6.2%)	45 (10.3%)	72 (16.6%)
Centre of Jeddah	14 (3.2%)	15 (3.4%)	29 (6.7%)
North of Jeddah	95 (21.8%)	205 (47.2%)	300 (69.1%)
East of Jeddah	9 (2.0%)	24 (5.5%)	33 (7.6%)
Education			
School (High school diploma or lower)	82 (18.8%)	117 (26.9%)	199 (45.9%)
College diploma	26 (5.9%)	93 (21.4%)	119 (27.4%)
Bachelor	20 (4.6%)	53 (12.2%)	73 (16.8%)
Masters or PhD	17 (3.9%)	26 (5.9%)	43 (9.9%)
Occupation			
Housewife	76 (17.5%)	122 (28.1%)	198 (45.6%)
Student	7 (1.6%)	22 (5.0%)	29 (6.7%)
Healthcare worker	7 (1.6%)	22 (5.0%)	29 (6.7%)
Others	55 (12.6%)	123 (28.3%)	178 (41%)
Monthly income			
<4,000 SR month	32 (7.3%)	104 (23.9%)	136 (31.3%)
Between 4,000 SR–10,000 SR/month	63 (14.5%)	96 (22.1%)	159 (36.6%)
>10,000 SR/month	50 (11.5%)	89 (20.5%)	139 (32%)
Heard about cervical cancer			
Yes/No	117 (26.9%)/28 (6.4%)	229 (52.7%)/60 (13.8%)	346 (79.7%)/88 (20.3%)
Perceived risk of developing cervical cancer			
Much below average	57 (13.1%)	143 (32.9%)	200 (46.1%)
Below average	38 (8.7%)	65 (14.9%)	103 (23.7%)
Average	36 (8.2%)	65 (14.9%)	101 (23.3%)
Above average	13 (2.9%)	9 (2.0%)	22 (5.1%)
Much above average	1 (0.2%)	7(1.6%)	8 (1.8%)
Heard about cervical cancer screening (Pap test)			
Yes	111 (25.5%)	172 (39.6%)	283 (65.2%)
No	34 (7.8%)	117 (26.9%)	151 (34.8%)
Source of information about cervical cancer screening (n = 283)			
Health care provider/doctor	65 (14.9%)	26 (5.9%)	91 (21%)
Brochures/posters	16 (3.6%)	37 (8.5%)	53 (12.2%)
Social media/TV	22 (5.0%)	81 (18.6%)	103 (23.7%)
Relative or friends	8 (1.8%)	28 (6.4%)	36 (8.3%)
Do you have a family doctor or (regularly visit primary health care centers)?			
Yes	41 (9.4%)	28 (6.4%)	69 (15.9%)
No	104 (23.9%)	261 (60.1%)	365 (84.1%)
Gender of family doctor (n = 69)			
Male	12 (2.7%)	7 (1.6%)	19 (4.4%)
Female	29 (6.6%)	21 (4.8%)	50 (11.5%)
Has the family doctor recommended Pap test?			
Yes	63 (14.5%)	14 (3.2%)	77 (17.7%)
No	82 (18.8%)	275 (63.3%)	357 (82.3%)
History of gynecological problem			
Yes	75 (17.2%)	95 (21.8%)	170 (39.2%)
No	70 (16.1%)	194 (44.7%)	264 (60.8%)
Previous gynecological examination			
Yes	128 (29.4%)	193 (44.4%)	321 (74%)
No	17 (3.9%)	96 (22.1%)	113 (26%)
Visit a gynecologist before			
Yes	138 (31.7%)	245 (56.4%)	383 (88.2%)
No	7 (1.6%)	44 (10.1%)	51 (11.8%)
Previous abortion			
Yes	70 (16.1%)	104 (23.9%)	174 (40.1%)
No	75 (17.2%)	185 (42.6%)	260 (59.9%)
Median number of children (IQR)	3 (2)	2 (3)	3 (3)

**Figure 1:**
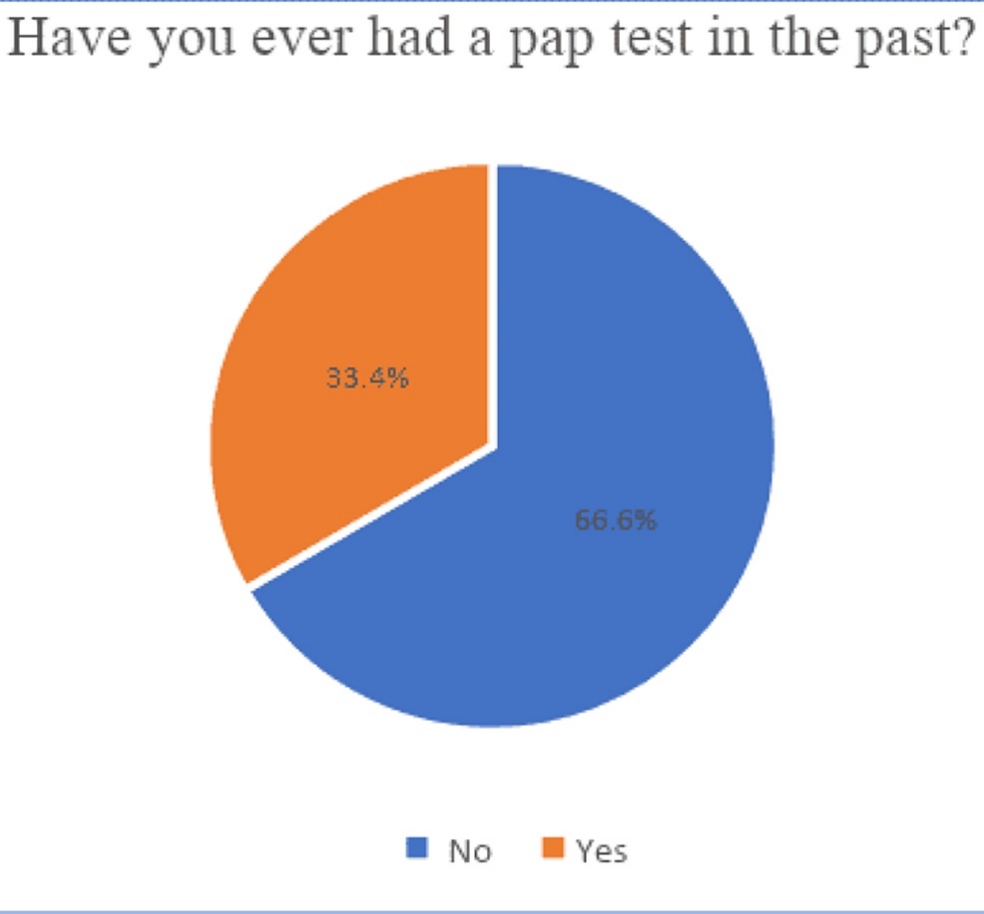
Distribution of having Pap test among the study participants

Among the 434 individuals who completed the survey during the study period, 145 (33.4%) had been screened for cervical cancer (have had a Pap test), whereas the majority, 289 (66.6%) of the participants, had never been screened. Figure 1 shows the distribution of the study participants based on the status of cervical cancer screening.

**Figure 2:**
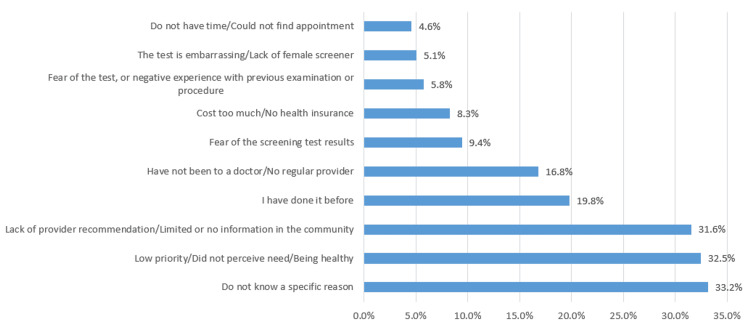
Barriers to having Pap test as reported by the participants

The 289 participants who had not had a Pap test were asked about the reasons or barriers. The most common reported answer (33.2%) was for unknown or unspecified reasons, followed by lack of perceived need due to being healthy (32.5%), and finally, lack of providers’ recommendations and limited availability of information about the test (31.6%). Figure 2 shows the barriers to cervical cancer screening as reported by participants who had never had a Pap test.

To determine predictors associated with the study outcome, undergoing cervical cancer screening, each of the demographic and predictor variables was tested independently using univariant binary regression. Increasing age showed an increased likelihood of screening. Compared to the reference age category (21-24 years), the age group 35-44 years had an odds ratio (OR) = 4.199 and p = 0.011, the age group 45-54 years had OR = 6.679 and p = 0.001, and the age group 55-65 years had OR = 8 and p = 0.001. In addition, education level had a significant association with screening status; in comparison with participants with high school education or less, participants with a diploma had OR = 0.399 and p = 0.001, and those with a Bachelor’s degree had OR = 0.538 and p = 0.039. Higher education levels (Masters or Ph.D.) were not significantly different from the reference group (p = 0.840). Higher monthly income was also found to be associated with participating in screening for cervical cancer. Participants with an income of 4000-10,000 SR/month had OR = 2.133 and p = 0.003, and those with an income more than 10,000 SR/month had OR = 1.826 and p = .025, in comparison with the lower-income participants. A non-significant association was found between cervical cancer screening status and the other demographic characteristics: marital status, nationality, and area of residence.

Although knowledge about cervical cancer was not a significant predictor (p = 0.723), the perceived risk of developing cervical cancer was significant. In comparison to women with very low perceived risk, women whose perceived risk was above average had OR = 3.624 (p = 0.005). The source of information about cervical cancer screening was also a significant predictor. Those who heard about Pap tests from sources other than the healthcare provider were significantly less likely to have a Pap test (p < 0.0001). The OR for brochures, social media and television, and relatives or friends were 0.173, 0.109, and 0.114, respectively. Having a family physician or regularly visiting a primary healthcare center was significantly associated with undergoing cervical cancer screening (OR = 3.675, p < 0.0001). However, the gender of the family physician had no significant association (p = 0.697).

When enquiring about gynecological history, women who were previously gynecologically compliant, who had undergone a gynecological examination, or those who had visited a gynecologist in the past, were more likely to have a Pap test (p < 0.0001), with OR 2.188, 3.745, and 3.541, respectively. Finally, the number of children was not significantly associated with screening status (p = 0.193), but having a previous abortion was (OR = 1.660, p = 0.014).

All variables that were significant or near significant were entered into a multivariable logistic regression model. The bivariate Pearson correlation was used to test the assumption that the variables included in the model did not have a significant correlation. A few variables were removed from the model as they had a significant correlation with other variables. The final multivariable logistic regression model included six variables (Table 2), all of which had a p < 0.1 in the univariate binomial regression and were not correlated. Participants’ age showed a significant association with the screening status (p = 0.037), where increasing age increased the likelihood of screening for cervical cancer. ORs and p-values for each age group are shown in detail in Table 2.

**Table 2:** Multivariate logistic regression estimates of factors associated with status of cervical cancer screening (had Pap test or not) . Variable(s) entered on step 1: var03, var07, var09, var18, var20, var23.

Factors		p-value	Odd Ratio (OR)	95% CI for OR	
				Lower	Upper
Age	21–24 years		Ref		
	25–34 years	.048	3.790	1.014	14.162
	35–44 years	.016	4.969	1.342	18.400
	45–54 years	.003	7.169	1.931	26.621
	55–65 years	.018	6.344	1.366	29.459
Education	School (primary, intermediate, high school)		Ref		
	Diploma	.226	.689	.377	1.259
	Bachelor (university or college)	.236	.653	.322	1.322
	Master or PhD	.649	1.205	.539	2.694
Income	<4000 SR/month		Ref		
	4000–10,000 SR/ month	.034	1.941	1.050	3.586
	>10,000 SR/month	.681	1.149	.593	2.226
Has your family doctor ever recommended a Pap test?	Yes	.000	14.432	7.218	28.856
Have you had any previous gynecological examinations?	Yes	.006	2.417	1.289	4.533
Have you had a previous abortion	Yes	.488	1.192	.726	1.958

Monthly income of 4,000-10,000 SR was significantly different to the reference group (<4,000 SR/month) (OR = 1.941, p = 0.034). The higher income group >10,000 SR/month was not significantly associated (p = 0.681). The recommendation of a Pap test by the family doctor was significantly associated with undergoing a Pap test (OR = 14.432, p < 0.0001). Similarly, undergoing a gynecological examination was a significant predictor (OR = 2.417, p = 0.006). Education level and a previous abortion were not significant predictors in the multivariable model (p = 0.372 and 0.488, respectively).

## Discussion

This study explored the utilization of cervical cancer screening and its predictors among women in Jeddah. A low prevalence of cervical cancer screening was found in the study subjects; only around one-third had undergone a Pap smear as screening for cervical cancer at some point in their lives. This is comparable to results from other national studies. In 2018 a descriptive cross-sectional study was conducted among 450 women living in Riyadh city and found that the pap test uptake was 26% [14]. Another study done in Al Qassim conclude that among 2220 participants, 1881 (84.7%) women had not undergone a Pap test [13]. International studies have also shown low screening rates. According to a cross-sectional study by Isabirye et al. in Uganda involving 845 women, only around 20% of women had undergone cervical cancer screening in their lives [10]. A cross-sectional study in Jordan found that only 31% of participants were screened for cervical cancer [15], which is comparable with these findings. A study in Kuwait reported that the percentage of participants that underwent a Pap test was around 24% [16]. Furthermore, in this study, a higher percentage of women aged 25-54 years participated in screening compared with women aged 21-24 years. This is consistent with a study among Kenyan women where younger age was associated with low screening rates [17]. This is most likely attributed to young females being more healthy and not seeking medical care frequently, unlike older females who may have other underlying conditions or comorbidities [16].

Previous studies have found that cervical cancer screening rates increase with educational level [17-18]. In this study, education level was associated with the outcome when tested for a single effect, but the association was not significant when tested for a combined effect. A higher income was associated with higher screening rates, in line with previous literature, which concluded employment and household wealth were associated with higher levels of screening [17-19]. This is also attributable to private health insurance of employed women [20].

Information about Pap tests and regularly visiting a family physician were significant predictors of screening (when tested for single effect), which is consistent with previous studies. The previously mentioned Kuwaiti study found that 77% of participants knew of screening tests, and the most common source of knowledge was a family doctor or a gynecologist (42%) [16]. Women with regular visits to primary health care or family physicians had higher rates of cervical cancer screening. This is consistent with other studies, which concluded that individuals who visited a healthcare facility in the last year were more likely to undergo cervical cancer screening [21-22].

The predictor with the largest effect on the screening status was the recommendation of a Pap test by the family doctor. This is consistent with previously published studies, showing that counseling and encouragement by the healthcare provider influence cervical cancer screening [15,23]. This finding supports the importance of family physicians’ visits for preventive health care and periodic health assessments. Similar to previous studies [23]. visiting a gynecologist, or undergoing a gynecological examination were significant predictors of cervical cancer screening. This finding is expected since many physicians consider Pap tests part of pelvic examination, and a Pap test is easier when patients are prepared.

Several factors were considered barriers by the participants, and some were agreed on by >30% of the sample (Figure 1). Notably, 33.2% did not have a specific reason blocking them from having a Pap test, which makes this option the most reported by the participants. Of the respondents, 32.5% did not perceive a need for a Pap test. This suggests that the level of awareness needs to be raised among the public, as well as the importance of patient counseling by doctors. Time or appointment unavailability was the least reported (4.6%) barrier by the participants.

 There are some limitations to this study. First, like all observational studies, it suggests an association only, but no causal relationship can be concluded. Second, the study was confined to the citizens of one city in Saudi Arabia, which may not be generalizable to all women in Saudi Arabia. Furthermore, data were collected electronically through social media platforms and, therefore, limited to people with social media or those with smartphones, which affects the generalizability of the results.

## Conclusions

Cervical cancer screening rate in the city of Jeddah is still relatively low. Larger multi-center studies on the Saudi population are needed to generalize the results to the Saudi population. The results also suggest that the factor with the largest impact is the recommendation of screening by the family physician, which stresses the important role of family doctors in counseling patients about screening. The study also suggests a need to promote cervical cancer screening through public campaigns to educate women about the early detection of cervical cancer and, at the same time, get a better understanding of the factors surrounding cervical cancer screening uptake.

## Appendices

Questionnaire to assess cervical cancer screening uptake and predictors among women in the city of Jeddah, Saudi Arabia

This questionnaire is designed exclusively for research purposes. It is targeting women, who are married (or have ever been married), aged 21- 65 years old, and reside in the city of Jeddah. All the data will be dealt with as highly confidential and will not be used for purposes other than this research. No participant could be identified in person. If you agree to participate in our study, please fill out the questionnaire. Kindly make sure to answer all questions.

I consent to participate in the study and fill out the survey

      Yes       

      No

------------------------

Demographic data:

1. Age

21-24 years

25-34 years

35-44 years

45-54 years

55-65 years

2. Marital status: 

Married

Divorced

Widowed

3. Nationality:

Saudi

Non-Saudi

4. Area of residence  (region of Jeddah):

South of Jeddah

Centre of Jeddah

North of Jeddah

East of Jeddah

5. Education:

School (primary, intermediate, high school)

Diploma

Bachelor (university or college)

Master or Ph.D.

6. Occupation:

Housewife

Student

Healthcare worker (doctor, nurse, physiotherapist, pharmacist, lab technician, ..etc)

Others

7. Income:

 Less than 4000 SR/ month

 4000-10,000 SR/ month

 More than 10,000 SR / month

------------------------

Screening status:

8. Have you ever had a pap test in the past:

  Yes

  No

9. Have you had a pap test in the past three years:

   Yes

   No

----------------------------

Predictors:

10. Have you heard about a disease called cervical cancer?﻿

Yes

No

11. Compared to other women of your age, what do you think your chances of getting cervical cancer are?

Much below average

Below average

Average

Above average

Much above average

12. Have you ever heard of cervical cancer screening (pap test)?

Yes

No

13. How did you learn about cervical cancer screening (Pap test)?

Healthcare provider/Doctor

Brochures/Posters

Social media/Television

Relative and/or friends

I have not heard about Pap test

14. Do you have a family doctor (or regularly visit a primary healthcare center)?

Yes

No

15. Is your family doctor:

Male

Female

I don’t have a family doctor

16. Has your family doctor ever recommended a Pap test?

Yes

No

17. Do you have any previous gynecological problems (abnormal bleeding or vaginal discharge, or others)

Yes

No

18. Have you done any previous gynecological examination:

Yes

No

19. Did you visit a gynecologist before:

Yes

No

20. How many children do you have: _______

21. Do you have any previous abortion:

Yes

No

_________________

Barriers

 22. If you have never undergone a Pap test, what are the reasons in your opinion (check all that apply)

I have done it before

Have not been to doctor/No regular provider

Lack of provider recommendation/Limited or no information in the community

 Cost too much/No health insurance

Do not have time/Could not find an appointment

Low priority/Did not perceive need/being healthy

Fear of the test, or negative experience with a previous examination or procedure

The test is embarrassing/Lack of female screener

Fear of the screening test results

Do not know a specific reason
